# In This Issue

**Published:** 2004

## SCREENING FOR ALCOHOL PROBLEMS: WHAT MAKES A TEST EFFECTIVE?

Screening for alcohol-related problems or alcohol use disorders is conducted in many different settings. Appropriately conducted screening tests can help clinicians better predict the probability that individual patients do or do not have a given disorder. Drs. Scott H. Stewart and Gerard J. Connors review some key characteristics that help to determine the effectiveness of screening tools, such as the test’s sensitivity, specificity, and positive and negative predictive values. The authors also discuss how other information available to physicians can be factored in to help determine a patient’s risk of alcohol-related problems. Finally, the authors emphasize that when choosing a screening tool, health care providers should ascertain that the tool is indeed appropriate for the specific population being screened and for the disorder(s) of interest (e.g., alcohol abuse, alcohol dependence, or both). (pp. 5–16)

## SCREENING IN GENERAL HEALTH CARE

Screening—a preventive service provided in general health care settings—allows clinicians to intervene early in the course of disease or to prevent the disease before it can develop. A mainstay of primary care, the practice of screening is firmly rooted in advances in public health made in the 1930s and 1940s, according to Dr. Marcia Russell. Over the next several decades, simple screens for conditions such as PKU showed their effectiveness in preventing disease, and the demand for screening tests and other types of preventive services grew. The need for increased prevention services created a problem for physicians, who often lacked knowledge about which services were most effective. To provide specific recommendations to practitioners about which services they should offer, the U.S. Preventive Services Task Force was developed. Dr. Russell uses the example of chronic hepatitis C infection to illustrate the criteria used by the U.S. Preventive Services Task Force in developing and evaluating screening and preventive services related to alcohol problems. (pp. 17–22)

## AN OVERVIEW OF NATIONAL ALCOHOL SCREENING DAY: TRENDS FROM 2001 TO 2003

National Alcohol Screening Day (NASD), the largest community-based intervention targeting alcohol misuse in the United States, was established in 1999 through a partnership between the National Institute on Alcohol Abuse and Alcoholism and the Substance Abuse and Mental Health Services Administration, with support from a variety of public and private organizations. The three primary objectives of NASD are to offer easily accessible anonymous alcohol screening to the general public at no charge, to refer for treatment those whom the screen identifies as consuming alcohol at unhealthy levels, and to educate the public about alcohol’s impact on general health. Mr. Matthew E. Dupre and Drs. Robert H. Aseltine, Jr., Gene V. Wallenstein, and Douglas G. Jacobs describe NASD’s implementation as well as trends in participation and results over the past 3 years. During this time, both the number of college and community organizations participating in NASD and the number of people being screened have increased significantly. Initial findings suggest that NASD is effective in motivating people who have unhealthy drinking patterns, and who have not been reached previously, to take the first step and go for screening; data from the past 3 years support this conclusion. (pp. 23–26)

## BIOMARKERS FOR ALCOHOL USE AND ABUSE – A SUMMARY

Clinicians working with alcohol abuse and alcoholism are always seeking new tools for evaluating and monitoring their patients’ alcohol use. Clinicians have several biochemical measurements available to objectively assess patients’ current or past alcohol use. None of the current biomarkers, however—including measures of various liver enzymes and blood volume—are ideal. Clinicians need to be able to obtain objective, quantitative information about a person’s current or historical alcohol consumption. Genetic information, including the patient’s risk of becoming alcohol dependent, also is important. In this article, Dr. Karen Peterson reviews standard biomarkers and several more experimental markers that hold promise for measuring acute alcohol consumption and relapse. Promising markers include alcohol byproducts such as acetaldehyde, ethyl glucuronide (EtG), and fatty acid ethyl esters (FAEE), as well as measures of sialic acid, a carbohydrate that appears to be altered in alcoholics. Some progress also has been made in finding markers that predict people’s genetic predisposition to alcoholism, such as genetic differences in several neurotransmitters, including beta-endorphin and gamma-glutamyltransferase (GABA). (pp. 30–37)

## BIOMARKERS OF ALCOHOL USE IN PREGNANCY

A significant percentage of women report drinking some alcohol during pregnancy, and risky drinking during pregnancy can result in a wide range of preventable birth defects, including fetal alcohol syndrome and fetal alcohol spectrum disorders. An estimated 1 percent of all live births show some alcohol-related prenatal damage. Currently no laboratory test is available that can identify and quantify prenatal alcohol use occurring over a protracted period. Clinicians commonly obtain maternal self-reports of drinking during pregnancy, but these measures may not be accurate. Many pregnant women are reluctant to disclose that they are or have been drinking alcohol during pregnancy, or their recall of how much and how often they drank may be unreliable. Developing effective biomarkers for prenatal alcohol use would provide the means to identify at-risk pregnancies and intervene with the aim of reducing alcohol-caused damage to the fetus. Drs. Cynthia F. Bearer, Joan M. Stoler, Janine D. Cook, with Dr. Siri J. Carpenter review the research on promising biomarkers for prenatal alcohol use. They describe a number of potentially useful biomarkers, including blood marker batteries that measure biochemical changes associated with alcohol use, fatty acid ethyl esters (FAEEs), and proteomic approaches to measuring the effects of alcohol use. (pp. 38–43)

## BRIEF INTERVENTIONS FOR ALCOHOL PROBLEMS

Brief interventions emphasize moderating alcohol consumption to sensible levels and eliminating binge drinking rather than advising abstinence. Drs. Anne Moyer and John W. Finney review studies of brief interventions to show how these approaches can be successful in a variety of audiences and settings. Several studies have supported the overall efficacy of brief interventions, particularly in primary care, although their long-term effectiveness may be limited. Research shows that these interventions appear to be cost-effective. Research also has shown that problems associated with drinking begin at levels of alcohol consumption much lower than those previously thought to warrant treatment, making brief interventions a potentially useful approach for people engaged in a variety of drinking behaviors. The authors describe how technology, such as computer programs and the Internet, can be used to implement brief interventions in busy emergency departments and overscheduled primary care offices. Technology also offers a means for training health care staff so that they feel comfortable providing interventions to patients, and for reducing the cost of providing interventions. (pp. 44–50)

